# Whole Genome Resequencing Reveals Natural Target Site Preferences of Transposable Elements in *Drosophila melanogaster*

**DOI:** 10.1371/journal.pone.0030008

**Published:** 2012-02-09

**Authors:** Raquel S. Linheiro, Casey M. Bergman

**Affiliations:** Faculty of Life Sciences, University of Manchester, Manchester, United Kingdom; University of California Riverside, United States of America

## Abstract

Transposable elements are mobile DNA sequences that integrate into host genomes using diverse mechanisms with varying degrees of target site specificity. While the target site preferences of some engineered transposable elements are well studied, the natural target preferences of most transposable elements are poorly characterized. Using population genomic resequencing data from 166 strains of *Drosophila melanogaster*, we identified over 8,000 new insertion sites not present in the reference genome sequence that we used to decode the natural target preferences of 22 families of transposable element in this species. We found that terminal inverted repeat transposon and long terminal repeat retrotransposon families present clade-specific target site duplications and target site sequence motifs. Additionally, we found that the sequence motifs at transposable element target sites are always palindromes that extend beyond the target site duplication. Our results demonstrate the utility of population genomics data for high-throughput inference of transposable element targeting preferences in the wild and establish general rules for terminal inverted repeat transposon and long terminal repeat retrotransposon target site selection in eukaryotic genomes.

## Introduction

Transposable elements (TEs) are mobile DNA sequences that can be found in virtually all organisms from prokaryotes to eukaryotes. TEs are considered as a major source of variability in evolution since the processes of insertion and excision can cause disruption of genes, chromosomal rearrangements, changes in genome size and other effects on the genome [1]. TEs can be categorized into two major classes according to their method of transposition: (i) those that transpose directly into the host genome *via* a DNA molecule (transposons), and (ii) those that transpose through an RNA intermediate (retrotransposons) [2]. The major group of transposons contain terminal inverted repeats (TIRs), whereas retrotransposons have two major subdivisions based on the presence or absence of long terminal repeats (LTRs) [3]. A characteristic mark of TE insertion in the genome is the presence of a target site duplication (TSD), which occurs upon TE integration as a result of staggered double-strand breaks at the target site [2]. TIR and LTR elements insert into target sites as a DNA-protein complex that are thought to cause a fixed length staggered cut that is characteristic of the TE family [2]. In contrast, transposition of non-LTR elements transposition leaves a variable length staggered cut in the genome that leads to a variable distribution of TSD lengths for a given family [4].

Understanding the molecular details of the target sites of TE integration is important for several reasons. First, understanding of TSD properties can provide further insight into the general process of transposition for a family or higher order taxonomic group of TEs. For example, analysis of the sequences around TSDs can reveal target site motifs (TSMs) that reflect the degree of structural [5] or sequence [6] specificity for TE insertion. This knowledge can be used to assess the potential insertion bias of TEs in genome-wide mutagenesis or evolutionary genomics studies. TSDs can also be used to characterize a new family of either TIR transposons or LTR retrotransposons [7], [8], since TSD length and sequence preferences for these types of element are thought to be conserved throughout the family. Finally, since TSDs delimit the extent of TE insertions in the genome, knowledge of TSD structure can be used to help annotate the location of TEs in genome sequences. For example, tools like LTRharvest [9], [10] use the TSD among other characteristics to identify new LTR insertions in the genome.

Properties of target sites are typically studied through the analysis of DNA sequences flanking TE insertions, which can be identified by spontaneous mutation [11], [12], [13], [14], artificial mutagenesis [5], [15], [16], [17], or in genomic sequences [8], [18], [19]. Despite providing useful insights into target site structure for a variety of TE families, these classical methods for target site analysis have some important limitations. For example, methods that rely on the analysis of spontaneous mutations or genome sequences are often based on small samples of insertions and do not allow analysis of the pre-integration target sequence, which is critical for accurate determination of TSD length and TSM sequence. Likewise, for methods that use artificially-induced transposition events, it is not usually known whether discovered TSDs or TSMs reflect those that would be generated by natural transposition events. As a consequence, TSDs and TSMs are only known for a limited number of TE families, and the general principles underlying target site structure and formation across broader clades of TEs in nature remain a mystery.

Here we develop a high-throughput approach to identify TSDs and TSMs based on the analysis of *de novo* TE insertions discovered using next-generation sequence data from whole genome shotgun (WGS) resequencing projects. All that is required for our method is a reference genome, a library of known TE sequences, and WGS data with reads long enough to include the start or end of an integrated TE and its unique genomic flanking sequence. We apply our approach to *D. melanogaster*, a species that has a broad range of previously characterized TE families that encompasses the diversity of TE types found in other eukaryotes [20]. Furthermore, TEs in *D. melanogaster* are generally polymorphic [21] and thus many additional TE insertions exist in natural populations beyond those observed in the reference genome. Moreover, a growing number of resequenced genomes are now available in *D. melanogaster* as a consequence of ongoing population genomics projects [22], [23]. Finally, well-studied TEs in *D. melanogaster* (such as the *P*-element) provide controls to test our system and to compare TSDs and TSMs inferred from natural insertions to those based on artificial insertions [5], [6].

Using resequencing data from 166 isofemale strains of *Drosophila melanogaster* produced by the *Drosophila* Genetic Reference Panel (DGRP) project [22], [23], we identified over 8,000 new TE insertion sites not present in the reference genome sequence [24] that we use to analyze properties of TSDs and TSMs for 22 families of TIR and LTR elements. By analyzing data gathered from both 454 and Illumina sequencing platforms, we show that different next generation sequencing platforms generally give consistent results in terms of *de novo* insertion site discovery. We found that TE families from the same clade present similar TSDs and TSMs, and that TSMs as a rule were palindromes that extended beyond the TSD. Furthermore, we were able to show that TSDs and TSMs previously identified from small samples or artificial mutagenesis experimental are comparable to those inferred from large datasets of natural transposition events. Together these results demonstrate that population genomic resequencing data can be used to rapidly discover TSDs and TSMs in a wild-type genomic context, allowing a better understanding of TE integration mechanisms in nature.

## Methods

### Identifying *de novo* TE insertions from whole genome shotgun sequences

Compressed fastq files from all accessions in the DGRP project were downloaded from the NCBI Short Read Archive and meta-data for each accession was used to concatenate reads from different accessions of the same DGRP strain. Reads were then given unique identifiers to account for the fact that pair-end reads from the same fragment do not have unique identifiers and converted into fasta files. We chose to analyze reads from paired-end runs as single-ended fragments since not all strains had paired-end data (including all 454 datasets) and our methods rely only on the contiguity of information contained within a single read.

**Figure 1:**
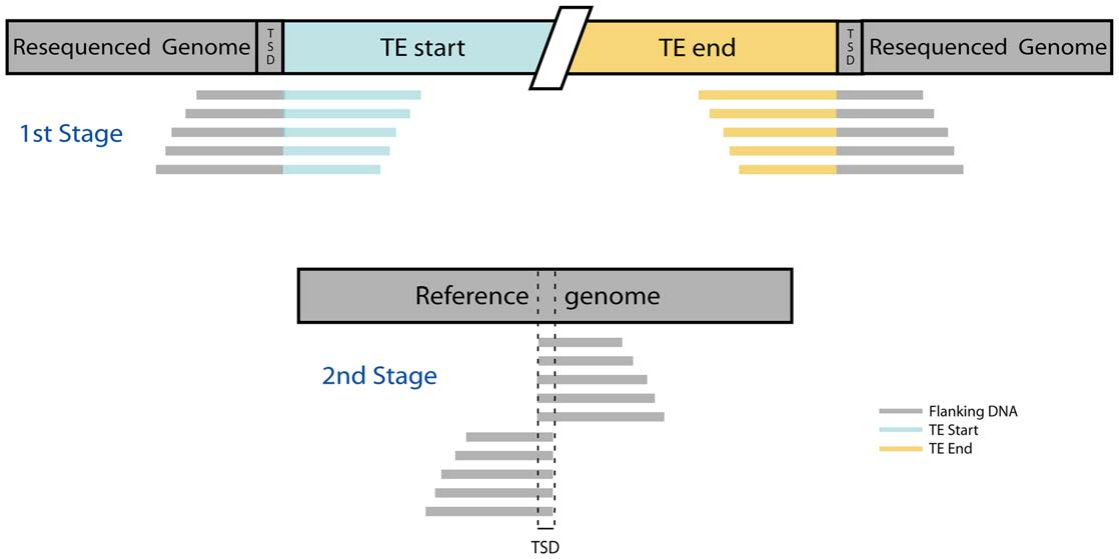
Overview of *de novo* TE insertion site mapping strategy. We detected *de novo* TE insertions using a two-stage process that relies on the presence of TSDs. In the first stage (top), unaligned and unassembled WGS sequence reads from a resequenced genome that has an integrated TE insertion were queried against a library of canonical TE sequences. Reads that span the junction of the start or end of TE and genomic flanking sequences are retained. In the second stage (bottom), the unique genomic DNA components of junction reads identified previously were aligned against the reference genome. The region of overlap between sets of junction reads that span the start and end of the same TE was used to define the TSD and orientation of *de novo* TE insertions.

We identified *de novo* TE insertions (i.e. insertions not present in the reference genome) from WGS resequencing reads using a two-stage selection processes (Figure 1). In both stages we used default settings of BLAT (version 34) [25], which imposed a minimum match length of 31 bp (tileSize = 11, stepSize = 11 and minMatch = 2; http://genome.ucsc.edu/FAQ/FAQblat.html#blat8). In the first stage, we used BLAT to query WGS reads against the FlyBase (version 9.4.2) fasta file of canonical sequences for 128 *D. melanogaster* TE families. We only kept reads whose best matches included the start (the first base of the 5′ end) or end (the last base of the 3′ end) of the TE query. If a read had two or more matches to different TEs, we discarded it if the spans were overlapping on the read and kept the best hit if they did not. The best matching TE was selected according to two criteria, the quality of the match and the length of the matching sequence. Better quality matches were defined as those with a lower number of blocks, gap bases and mismatching bases. Matches were discarded if they had more than one block, gap, or mismatch for every 20 bp of target and query sequences. When a WGS read had two or more hits for the same TE family, we retained the match with the best quality and length. When a read matched the start and end of a TE equally well, we randomly picked one end. When a match was indistinguishable between a start/end and the middle of a TE, we selected for the start/end match.

During the second stage, we mapped reads that included the starts or ends of TEs identified in stage one to the Release 5 *D. melanogaster* genome sequence using default BLAT settings. We then selected for mapped reads with one or fewer mismatch in 20 for both the read and reference genome sequences. Reads were retained if a match to the reference genome or TE was included the beginning or end of the read. These sequences also had to match the reference TE start/end exactly where the genomic region begins or *vice versa*. Selected reads could only map to the genome in one location: if there was ambiguity about the exact location in the genome of a sequence with the same criterion, the read was discarded. This approach only identifies *de novo* TE insertions with both termini present in unique regions of the genome, and thus new insertions of 5′ truncated non-LTR elements, severely internally truncated TIR elements and insertions into repetitive DNA will not be identified by this method.

### Identification of target site duplications

Our approach to TSD identification relies on identifying *de novo* TE insertion sites in resequencing data that are not present in the reference genome, so we can compare the pre-integration sequence in the reference genome to the post-integration sequence in the resequenced genome. To find TSDs of *de novo* insertions, we identified sets of mapped reads that (i) passed our two-stage filtering procedures above, (ii) matched the same reference TE, and (iii) had distances between the start coordinate of one read and the end coordinate of the next read found sequentially in the genome that overlapped by less than or equal to 20 bp. This overlap distance defines the TSD (see Figure 1). We predicted a TSD for a *de novo* TE insertion if there were one or more reads supporting each side of the overlap region. To automatically define the optimal TSD length for each family, we then identified the mode of the distribution of TSD lengths of individual insertions for TE families with greater than eight insertions. This TSD identification strategy selects for TE families with a fixed TSD length, which is only applicable for LTR and TIR elements. As a consequence of the requirements for a fixed-length TSD and inclusion of both termini of a full-length TE in our read selection procedures (see above), we excluded non-LTR elements from our analysis in this study. We note that the maximal TSD length that we can discover using the current approach is 20 bp. However our results show that this cutoff exceeds the optimal TSD width of most TE families in *D. melanogaster*, and this arbitrary parameter could be adjusted for other species.

### Analysis of target site motifs

TSMs were constructed by concatenating sequences extending ±15 bp around the TSD from the non-redundant set of insertion sites for each family into a multiple alignment. Sequences of insertion sites on the negative strand were reverse complemented before inclusion in the alignment. Position frequency matrices were automatically created in R (version 2.9.1) [26] and were then used to create sequence logos [27] using a custom implementation in R. High information content nucleotides positions typically did not extend beyond ±3 bp around the TSD, and thus this window was chose to plot logos.

## Results

### Next generation population genomic resequencing data provide an abundant source of *de novo* TE insertions

In order to find *de novo* TEs insertion sites in the *D. melanogaster* genome for TSD and TSM discovery, we identified “junction reads” (also known as “split reads” [28]) that contain both unique genomic and repetitive TE sequences in a single sequencing read. In brief, we first aligned 454 and Illumina sequencing reads from the DRGP project to the set of known *D. melanogaster* TE canonical sequences. Reads that mapped to the start or end of the reference TE were selected and subsequently mapped against the *D. melanogaster* reference genome to find the TE insertion site and TSD (see Figure 1 and Materials and Methods for further details). For the 454 data, we processed 209,979,997 reads from a total of 34 strains and retained 44,254 reads (0.021% of the total) across 34 strains that included a TE start/end for a TIR or LTR element that could be mapped to the reference genome (File S1). For the Illumina data we processed 7,835,189,604 reads from a total of 176 strains and retained 65,488 reads (0.00084% of the total) across 166 strains that uniquely matched a start or end of a TE for a TIR and LTR element that could be mapped to the reference genome (File S2). We note that 25 strains with reads supporting *de novo* insertions were sequenced by both platforms (see below).

Since our focus is on discovering new target sites in the genome, we only consider non-redundant insertion sites at the same position in the genome on the same strand regardless of their allele frequency in the set of DGRP strains, unless otherwise noted. In contrast to the typical approach of annotating TE insertions that are not in the reference genome to a single base location, we annotated *de novo* TE insertion by their TSD span, since *de novo* TE insertions can be annotated ambiguously at the 5′ or 3′ end to different genomic locations under a single base annotation scheme [28], [29]. Across all strains, we predicted 3,386 *de novo* TE insertion sites using 454 reads and 8,024 *de novo* TE insertion sites using Illumina reads (Table 1). Predicted *de novo* insertions were supported by a median of 12 and six reads, respectively, in the 454 and Illumina datasets. Genomic locations of *de novo* insertion sites from the 454 and Illumina datasets are available in File S3 and File S4, respectively.

**Table 1:** Number of *de novo* TE insertions identified in resequencing data from the DGRP project.

Order	Superfamily	# Insertions 454	# Families 454	# Insertions Illumina	# Families Illumina
TIR	*hAT*	437	1	1,198	1
TIR	*P*	465	2	1,505	2
TIR	*Pogo*	245	1	895	1
TIR	*Tc1*	12	1	25	1
TIR	*Transib*	153	2	540	2
TIR	All	1,312	7	4,163	7
LTR	*Copia*	156	3	1	1
LTR	*Gypsy*	1,569	24	3,445	25
LTR	*Pao*	349	4	415	5
LTR	All	2,074	31	3,861	31
TIR+LTR	All	3,386	38	8,024	38

Shown are numbers of non-redundant TE insertion sites and families discovered for different orders and superfamilies of TE based on 454 or Illumina resequencing data.

In total, we found *de novo* TE insertions for 38 different families in both platforms (Table 1). For TIR elements, both platforms identified the same set of seven TE families. For LTR elements, we identified *de novo* insertions for 31 families on both platforms, but only 23 of these families were common to both platforms. Eight TE families were found exclusively in the 454 data (*1731*, *copia*, *diver*, *flea*, *HMS-Beagle2*, *invader2*, *Springer*, *Stalker4*) or in the Illumina data (*gypsy12*, *invader3*, *invader6*, *rooA*, *aurora-element*, *Tirant*, *rover*, *ZAM*). With the exception of the *copia* family in the 454 data (n = 153), all LTR families that were detected in only one platform had fewer than five *de novo* insertions. Thus, we conclude that discovery of TE insertions for a given family is consistent among 454 and Illumina platforms, except when the number of *de novo* sites for a family is low.

**Figure 2:**
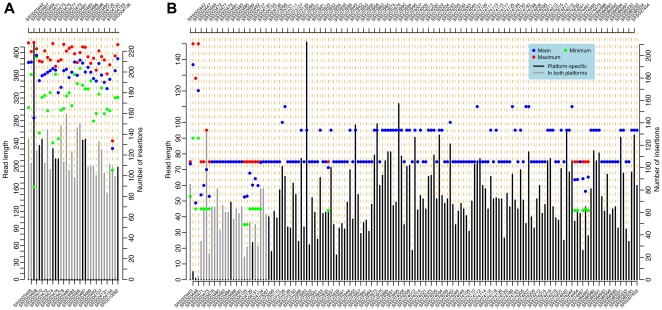
Read length and number of insertions per strain for DGRP resequencing datasets. Summary of data from the 454 platform (A) and the Illumina platform (B). Points represent the maximum, minimum and mean read length for each strains (scale bar on left). Bars represent the total number of elements identified per strain (scale bar on right). Gray bars represent the number of insertions for strains sequenced by both 454 and Illumina, and black bars represent the number of insertions from strains with platform-specific sequence data. Strain identifiers labeled alternately on the top and bottom of the graph.

We were able to find *de novo* insertion sites in all 34 strains sequenced by the 454 platform. For these strains, we identified a minimum of 83 new insertions per strain (Figure 2 A). In contrast, we were able to identify insertion sites for only 166 out of the 176 strains sequenced by the Illumina platform. The ten strains with no detectable insertions (NCBI Short Read Archive accessions: SRS003467, SRS003469, SRS003470, SRS003474, SRS003475, SRS003476, SRS003486, SRS003487, SRS004126, SRS004137) had read lengths less than 64 bp long. For the remaining 166 strains sequence sequenced by the Illumina platform with data of length greater then 75 bp, we identified a minimum of 20 new insertions per strain with three exceptions that had fewer than eight new insertions per strain (Figure 2 B). Three strains had fewer than eight new insertions (SRS003443, SRS003447 and SRS003448) and showed a very unusual pattern of quality scores across the length of the read relative to the expected decline in quality towards the end of the read (Figure S1). The pattern of quality scores in these strains was consistent with an adaptor being present in the middle of the sequence [30], which can occur if two reads have been concatenated into one.

### Insertion site predictions based on 454 and Illumina resequencing data are consistent but not comprehensive

To better understand differences in TE insertion site predictions on the 454 and Illumina platforms, we compared insertion sites for the 25 strains that had been sequenced on both platforms (SRS003442, SRS003448, SRS003468, SRS003471, SRS003472, SRS003473, SRS003477, SRS003478, SRS003479, SRS003480, SRS003481, SRS003482, SRS003483, SRS003485, SRS003488, SRS003489, SRS003490, SRS003492, SRS004125, SRS004127, SRS004130, SRS004131, SRS004133, SRS004134 and SRS004136). For this analysis, we restricted our focus to the 22 most abundant families (defined as those with eight or more insertion sites in the Illumina dataset) in an attempt to mitigate against random effects of small sample sizes. For these families, we counted the number of times each predicted insertion site was seen in the same location in both platforms in the same strain. A summary of this analysis by family is shown in Table 2 and data for individual insertion sites can be found in File S5.

**Table 2:** Comparison of *de novo* TE insertions in 25 strains sequenced by both 454 and Illumina platforms.

Order	Superfamily	Family	Non-redundant insertion sites in 454	Non-redundant insertion sites in Illumina	Same location	Same location and TSD	Same location, TSD and strand
TIR	*hAT*	*hobo*	323	192	173	172	172
TIR	*P*	*1360*	65	54	38	38	37
TIR	*P*	*P*-element	258	150	134	133	133
TIR	*Pogo*	*pogo*	176	160	112	112	112
TIR	*Tc1*	*S*-element	9	1	1	1	1
TIR	*Transib*	*hopper*	115	80	50	50	48
LTR	*Gypsy*	*297*	10	5	4	4	4
LTR	*Gypsy*	*412*	136	75	65	64	64
LTR	*Gypsy*	*blood*	115	62	58	58	58
LTR	*Gypsy*	*Burdock*	146	81	79	78	78
LTR	*Gypsy*	*gtwin*	6	3	2	2	2
LTR	*Gypsy*	*gypsy*	27	19	15	15	15
LTR	*Gypsy*	*HMS-Beagle*	116	59	56	56	56
LTR	*Gypsy*	*mdg1*	151	9	9	9	9
LTR	*Gypsy*	*opus*	267	130	122	118	118
LTR	*Gypsy*	*Quasimodo*	4	2	2	2	2
LTR	*Gypsy*	*Stalker2*	40	15	15	15	15
LTR	*Gypsy*	*Tabor*	58	26	24	24	24
LTR	*Gypsy*	*Transpac*	50	36	31	31	31
LTR	*Pao*	*3S18*	33	15	15	14	14
LTR	*Pao*	*Max*-element	41	18	18	18	18
LTR	*Pao*	*roo*	180	19	15	15	15
TIR+LTR	All	All	2,326	1,211	1,038	1,029	1,026

Data is shown only for the most abundant TE families (those with eight or more insertions).

Overall, we found 2,326 insertion sites in the 454 data and 1,211 insertion sites in the Illumina data for these 25 strains. More insertion sites were also predicted per strain for the 454 data than for Illumina data for each family individually (Table 2). Higher numbers of insertions per strain in the 454 dataset are likely to arise from increased read length (median: 365 bp for 454; 75 bp for Illumina) rather than increased sequencing depth (median: 18× for 454; 26× for Illumina). The vast majority of the Illumina insertion sites were found in the 454 dataset in the exact same location with the same TSD and strand (1,026/1,211, 84.7%). In contrast, less than half (1,026/2,326, 44.1%) of the 454 insertions were supported exactly by an insertion from the Illumina dataset. Only a very small number of insertion sites were predicted to be in the same location and orientation but with a different TSD length (n = 9), or in the same location with the same TSD but on the opposite strand (n = 3). Differences in predicted TSD length or orientation may arise from inaccuracies in our insertion detection procedures or different types of sequencing errors generated by the different platforms. We note that the three insertions predicted to be in the same location but on opposite strands were from transposon families (*1360* and *hopper*) with terminal inverted repeats, which may have caused the orientation differences. Regardless of the source of these slight discrepancies, these data clearly indicate that, where data are available on both platforms for a given insertion site, they overwhelmingly yield consistent information about the identity, location and orientation of a *de novo* insertion. Assuming consistency is a measure of accuracy, we conclude that both 454 and Illumina platforms can be used to generate high quality *de novo* TE insertion site data in *D. melanogaster*. However, even at the average depth of sequencing coverage for a given strain studied here, a substantial number of TE insertions are detected by only one of the two sequencing platforms and thus neither dataset provides a comprehensive map of TE insertion sites in these strains using our current bioinformatic methods.

Despite the fact the 454 data provided more insertions per strain, we chose to base our subsequent analysis of TSDs and TSMs on the Illumina data since this platform had many more strains available and therefore provided a greater number of insertion sites overall (Table 1). Using the 166 strains of *D. melanogaster* that generated insertion site predictions from Illumina data, we were able to extract 8,024 non-redundant *de novo* TE insertions sites from 38 families, with each strain contributing on average 48.3 insertion sites. The TIR transposon order generated the highest number of *de novo* insertions with 4,163 insertion sites spread throughout five superfamilies and seven families (Table 1). The LTR retrotransposon order generated a total of 3,861 *de novo* insertion sites from three different superfamilies and 31 different families. The LTR superfamily with the highest number of insertions was *Gypsy* with 3,445 insertion sites in 25 different families. As shown in Table 3, the TIR transposon family with the greatest number of new insertion sites is the *P*-element (n = 1,226 insertion sites), a TE family that is not present in the reference genome sequence [20]. The LTR family with the greatest number of *de novo* insertion sites was the *opus* element (n = 1,030 insertion sites), which is moderately abundant in the reference genome sequence [20].

**Table 3:** Optimal TSD length and number of *de novo* insertion sites based on Illumina data.

Order	Superfamily	Family	Modal TSD length	Insertion sites	Insertion sites with modal TSD length	% Insertion sites with modal TSD length
TIR	*hAT*	*hobo*	8	1,198	1,196	99.83
TIR	*P*	*1360*	7	279	274	98.21
TIR	*P*	*P*-element	8	1,226	1,207	98.45
TIR	*Pogo*	*pogo*	2	895	883	98.66
TIR	*Tc1*	*S*-element	2	25	25	100
TIR	*Transib*	*hopper*	5	533	532	99.81
TIR	*Transib*	*transib2*	5	7	7	100
LTR	*Copia*	*Dm88*	3	1	1	100
LTR	*Gypsy*	*297*	4	19	18	94.74
LTR	*Gypsy*	*412*	4	498	494	99.20
LTR	*Gypsy*	*accord*	4	3	3	100
LTR	*Gypsy*	*blood*	4	378	376	99.47
LTR	*Gypsy*	*Burdock*	4	481	471	97.92
LTR	*Gypsy*	*gtwin*	4	19	18	94.74
LTR	*Gypsy*	*gypsy*	4	92	92	100
LTR	*Gypsy*	*gypsy12*	4	1	1	100
LTR	*Gypsy*	*gypsy2*	4	2	2	100
LTR	*Gypsy*	*gypsy5*	4	6	6	100
LTR	*Gypsy*	*HMS-Beagle*	4	320	311	97.19
LTR	*Gypsy*	*Idefix*	5	1	1	100
LTR	*Gypsy*	*invader3*	4	1	1	100
LTR	*Gypsy*	*invader6*	4	1	1	100
LTR	*Gypsy*	*mdg1*	4	146	146	100
LTR	*Gypsy*	*mdg3*	4	5	5	100
LTR	*Gypsy*	*micropia*	4	1	1	100
LTR	*Gypsy*	*opus*	4	1,030	976	94.76
LTR	*Gypsy*	*Quasimodo*	4	9	8	88.89
LTR	*Gypsy*	*rover*	4	3	3	100
LTR	*Gypsy*	*Stalker2*	4	84	82	97.62
LTR	*Gypsy*	*Tabor*	4	138	138	100
LTR	*Gypsy*	*Tirant*	2	2	2	100
LTR	*Gypsy*	*Transpac*	4	202	202	100
LTR	*Gypsy*	*ZAM*	4	3	3	100
LTR	*Pao*	*3S18*	5	119	113	94.96
LTR	*Pao*	*aurora*-element	17–18	2	2	100
LTR	*Pao*	*Max*-element	5	100	96	96.00
LTR	*Pao*	*roo*	5	193	182	94.30
LTR	*Pao*	*rooA*	13	1	1	100

Families with fewer than eight insertion sites were excluded from further analyses of TSD and TSM structure, but often show similar modal TSD length to related TE families.

### TSDs have a characteristic length for TIR and LTR families and clades

We plotted the frequency distribution of TSD lengths for individual TE insertions from each of these 38 TE families in order to infer the optimal TSD length for the family. We note for this analysis we used TE insertion site predictions from all strains, since the TSD length predicted for a given insertion site in one strain is independent of other predicted insertion sites, even at the same location in a different strain. For 36 families we observed a single major peak in TSD length of less than 10 bp (Figure S2). The exceptions to this rule are for *rooA* and *aurora*-element, which have only one or two *de novo* insertion sites, respectively. Although the modal TSD length was typically shared by >95% of insertions from a family, we did observe some cases in which the TSD was different from the majority (Table 3, Figure S2). These cases represented a minority of the total number of predicted TSDs (1.8%) and were typically only ±1 bp from the optimal TSD for most elements with the exception of *opus*, which generated alternative TSD ±2 bp from the optimal TSD length. These low-frequency variant TSDs may represent real variation in TSD length, sequencing error, or artifacts of our TSD detection methods.

To draw general conclusions about target site properties, we focused on the 22 families for which we found eight or more *de novo* TE insertion sites (Table 3). From this subset of families, we find (i) that all TSDs were less than 10 bp and (ii) that TSDs of TE families from the same clade typically showed similarities in length. LTR elements from the *Gypsy* group presented a strong preference for a TSD of four bp (see also [19]), and those from the *Pao* group families had a TSD of five bp. However, optimal TSD lengths from TEs in the *P*-element group did not agree with each other, with the *P*-element having an optimal TSD length of eight bp but the *1360* element displaying an optimal TSD length of seven bp. We also note that families with fewer *de novo* insertions than our arbitrary cutoff of eight typically shared TSD length with the rest of their respective clade, suggesting that data on TSD length from some low sample size families are also meaningful. Exceptions to this rule, however, are observed for *Idefix*, *Tirant*, *rooA* and *aurora*-element, all of which have only one or two *de novo* insertion sites in our data set.

### TSMs for TIR and LTR elements are palindromes that extend the TSD and follow phylogenetic relationships of TE families

**Figure 3:**
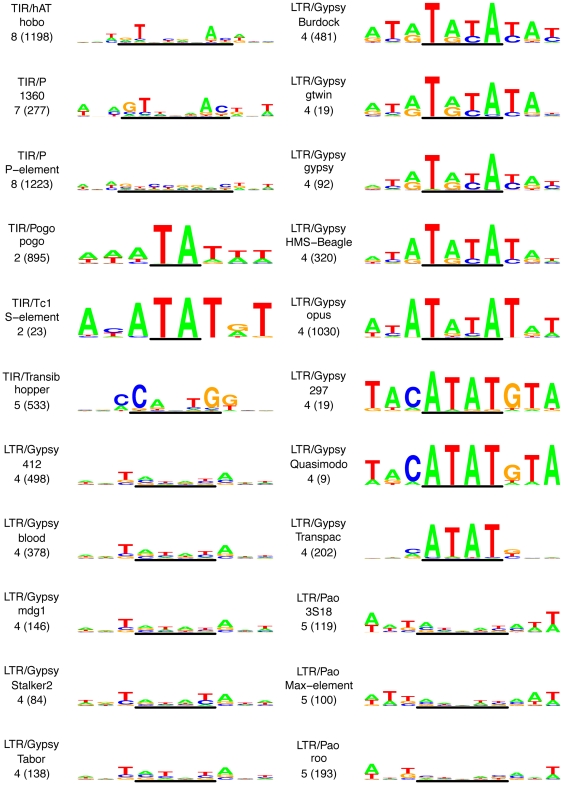
Sequence logos for target site motifs of 22 *D. melanogaster* TIR and LTR families. Predicted TSMs plotted as sequence logos for sequences ±3 bp around the TSD for TE families with eight or more insertion sites. Plots are organized by order (TIR then LTR) and superfamily, and are labeled with order/superfamily, family name, predicted TSD length, and total number of insertion sites (in parentheses) in the top right corner. The y-axis is the same for all the logos and ranges from a bit score of zero to two. The line below the logo represents the TSD.

To identify sequence motifs associated with the target site, we aligned the TSD and flanking sequences for the 22 TE families with eight or more *de novo* insertion sites and produced sequence logos that represent the nucleotide usage at each position in the TSD and its flanking regions (Figure 3). These target site motifs (TSMs) represent the degree of target specificity a TE has for insertion sites in the genome and can in principle extend beyond the TSD, as has been shown previously for the *P*-element [5], [6]. In general, TE families with a high number of *de novo* insertion sites did not necessarily lead to a high information content TSM: families with just 25 insertion sites could generate a high information content TSM (e.g. *S*-element) while families with over 100 insertion sites result in a very degenerate motif (e.g. *412*). Additionally, the highest information content positions of a TSM were not always inside the TSD (e.g. *Stalker2*). TSMs range in length from seven bp (*hopper*) to 14 bp (*hobo* and *P*-element) and extend beyond the TSD by up to three bp. Consistent with their palindromic nature, families with odd-length TSDs typically have the lowest information content nucleotide of the TSM at the center of the TSD. Intriguingly, TSMs for all families from both the TIR and LTR orders showed two common properties: (i) a TSM that extends beyond the TSD and (ii) a preference for a palindromic motif. TSMs also showed a general tendency to be AT-rich for all LTR families and all but one TIR family (Figure 3, File S6). However, since the *hopper* family showed a clear preference for a GC-rich TSM, we cannot conclude that AT-richness is a strict rule for TSMs in TIR or all TEs in general.

As with TSD length, TEs from the same clade showed a similarity in their TSMs. For TIR elements from the *P*-element group there was a tendency to have an ANAGT motif on the 5′ half and an ACTNT motif on the 3′ half of the TSM. LTR elements from the *Pao* group all share a relatively low information content motif characterized by an AWTAWNWTAWT motif. TSMs from the *Gypsy* superfamily appear to fall into three discrete subgroups, which fall clearly along established phylogenetic lineages represented by the *412*, *gyspy* and *Transpac* families [8], [19], [31]. The TSM from the *412* clade (including the *412*, *blood*, *mdg1*, *Stalker2* and *Tabor* families analyzed here) contains a low information content ATAT motif spanning the TSD flanked by T and A on the 5′ and 3′ ends, respectively. The TSM from the *gypsy* clade (including *Burdock*, *gtwin*, *gypsy*, *HMS-Beagle* and *opus*) contains a central high information content TATA motif spanning the TSD and is flanked by A and T on the 5′ and 3′ ends, respectively. Finally, the TSM from the *Idefix* clade (including *297*, *Quasimodo* and *Transpac*) contains a central high information content ATAT motif spanning the TSD and is flanked by C and G on the 5′ and 3′ ends, respectively. We note that *Transpac* is the most divergent member of the *Idefix* clade in previously published phylogenies of LTR elements in *D. melanogaster* [8], [19] and also presents a divergent TSM relative to other members of the *Idefix* clade in our data as well. In the context of the wider phylogenetic relationships of the *Pao* and *Gypsy* clades, which can be represented in Newick format as (*Pao*,(*412*,(*gypsy*, *Idefix*))) [8], [19], [31], our data imply both an increase in target site specificity during the evolution of the more derived *gypsy* and *Idefix* clades, and at least one transition from an ATAT to a TATA core TSM. The latter transition may have been facilitated by a simple shift in the preferred target half-site, since the inferred ancestral state ATATAT (core TSM underlined), represented by the *412* clade, is only a ±1 base pair edit from the derived state TATATA, represented by the *gypsy* clade.

### TSDs and TSMs discovered using population genomic data are consistent with previous studies

A large body of information on the target site preferences of different TE families has been amassed in *D. melanogaster* based on data from spontaneous mutations, artificial mutagenesis, and genomic sequences [5], [6], [7], [8], [15], [19], [32], [33], [34], [35], [36], [37]. To assess the reliability of using high-throughput population genomic data from next-generation resequencing projects to study TSD and TSM properties, we compared our results for the 22 families with eight or more insertions to those based on previous studies that use these other sources of sequence information. Our results are consistent with previous data for 19 families that we could find published evidence about TSD length (Table 4). For two families (*pogo* and *412*), we could resolve previous ambiguities about TSD length and for an additional three families (*Stalker2*, *3S18* and *Max*-element) we generated entirely novel information about TSD length. We also compared our TSMs (converted to consensus sequence form) with previously published data on TSM for these 22 families (Table 4). As with TSD length, our TSM results based on population genomic data were broadly consistent with results based on other sources of evidence. However, we were able to generate more refined TSMs with either an extended motif length or less ambiguity for the vast majority (19/22, 86.4%) of TE families.

**Table 4:** Comparison of TSDs and TSMs identified in this study with previously published results.

Family	TSD (this study)	TSD (previous studies)	TSM (this study)	TSM (previous studies)	Reference
*hobo*	8	8	GTNCGNAC	NTNNNNAN	[32]
*1360*	7	7	GTTNAAC	KTNBWAB	[33]
*P*-element	8	8	GTCCGGAC	GTCCGGAC	[5], [6]
*pogo*	2	2 or 0	TA	TA	[15]
*S*-element	2	2	TA	AT	[34]
*hopper*	5	5	CCANTGG	n.a.	[35]
*transib2*	5	5	CCANTGG	CABHG	[7]
*297*	4	4	ATAT	ATAT	[19], [36]
*412*	4	4–6	ATAT	WKRK/NNAN	[8], [19]
*blood*	4	4	ATAT	RKAS/NNAN	[8], [19]
*Burdock*	4	4	TATA	TATA/TRYA	[8], [19]
*gtwin*	4	4	TRTA	TGTA/TRYA	[8]
*gypsy*	4	4	TRYA	TRYA	[19]
*HMS-Beagle*	4	4	TATA	TRTA/TRYA	[8], [19]
*mdg1*	4	4	ATAT	CTAC/NNAN	[8], [19]
*opus*	4	4	TATA	TANA/TRYA	[19], [37]
*Stalker2*	4	n.a.	ATAT	n.a.	n.a.
*Tabor*	4	4	ATNT	MMKS	[8]
*Transpac*	4	4	ATAT	ATAT	[19]
*3S18*	5	n.a.	ATNAT	n.a.	n.a.
*Max*-element	5	n.a.	AANTT	n.a.	n.a.
*roo*	5	5	CTNAC	VWWAY	[19], [35]

For the only TE family (the *P*-element) that had previously been inferred from a very large sample size (>10,000 insertion sites) [5], [6], we found identical TSD length and very similar TSM using population genomic and artificial mutagenesis data. Moreover, at the individual insertion site level, we found a surprising degree of overlap between artificially generated and naturally-occurring *P*-element insertions, when artificial *P*-element insertions from the *D. melanogaster* release 5.40 genome annotation are converted from the single-base to the TSD-based coordinates framework used here. Specifically, we find that 178 of the 1,226 naturally occurring *P*-element insertions identified in the Illumina dataset insert into the exactly the same genomic location (same coordinates and orientation) as insertions derived from *P*-element mutategensis, suggesting a high degree of fidelity for *P*-element target site selection as well accurate mapping of both artificial and natural insertions. Thus, we conclude that inferences based on population genomic data from next-generation resequencing projects are compatible with classical approaches to infer TSD and TSM properties, including those based on artificial mutagenesis experiments.

## Discussion

Here we show that WGS data from next generation resequencing projects can successfully be used to identify large samples of *de novo* insertions in order to discover properties of TE target sites in *D. melanogaster*. Assuming results for the families studied here can be generalized to other TE families, the major biological findings of this work are: (i) TSDs for TIR and LTR elements are less than 10 bp in length, (ii) TSD length for TIR and LTR elements are shared by related TE families in the same clade, (iii) TSMs for TIR and LTR elements are palindromes, and (iv) target sequence preferences for TIR and LTR element-encoded TSMs extend beyond the limits of the TSD. We believe these general conclusions about TIR and LTR target site preferences are robust for several reasons. First, for strains of *D. melanogaster* that have been independently sequenced using 454 and Illumina technologies, the insertion location, orientation and TSD are highly consistent among different platforms (Table 2). Thus, it is unlikely that the fundamental data used here to infer properties of TE insertion are heavily biased by the platform-specific sequencing errors. Second, our results based on population genomic data from wild-type flies is consistent with previous findings in *D. melanogaster* based on spontaneous and artificially generation mutations in lab strains (Table 4). This reproducibility across data types reciprocally implies that the inferences about TSD and TSM properties from both large-scale population genomic and classical data are reliable. Finally, we observe consistent phylogenetic signals in TSD length and TSM properties among related clades of TE families that are not predefined by constraints in our methodology and can only arise by common biological processes.

Our use of next-generation sequence data to study the details of target site preferences joins a growing number of applications that attempt to identify TE insertion mutations based on targeted or whole-genome resequencing. Broadly speaking, the aims of these previous techniques fall into two major classes: (i) genome-wide screens for insertions in DNA pools from a single TE family induced by artificial mutagenesis to identify genomic regions that are essential for growth in bacteria [38], [39], [40], [41] or tumors [42], [43], [44], and (ii) genome-wide screens in individuals/strains for spontaneous insertions from one or more TE family to study population genomics and genome evolution [45], [46], [47], [48]. The aim of our method for TE insertion discovery differs from these previous methods in that our approach is designed to reveal the mechanistic details of transposon insertion site preferences. As such, our approach employs stringent filtering to identify only well-supported *de novo* insertion sites, and attempts to annotate insertions at exact nucleotide-level resolution rather than provide a comprehensive map of all TE insertions in all strains.

In terms of studying TE insertion site preferences, our next-generation sequencing based population-genomic approach has many advantages over traditional methods. Our method can be applied in any species with active TEs, requires no artificial mutagenesis, is high-throughput and fully automated, generates TSD and TSM information simultaneously for all active TE families, uses a common biological data source and consistent computational methods for all TE families studied, allows direct comparison of pre-integration and post-integration genomic sequences, is based on naturally-occurring mutational events, and identifies the exact breakpoints of TE integration in the genome. Nevertheless, there are several key limitations with our TE insertion site discovery approach that prevent comprehensive application to all TE families and for use in other applications (e.g. population genetics). First, our method requires both termini of a full-length element to be present for a *de novo* insertion to be detected. Thus, we cannot identify incomplete *de novo* TE insertions such as 5′ truncated non-LTR retrotransposons. While our method can find full-length non-LTR elements, the variable TSD length of these TEs prevented automated inference of optimal TSD length for downstream filtering and TSM inference, which is why they were excluded from this study. Second, we require TE-junction information to be contained in a single read and our sequence similarity thresholds effectively require ∼30 bp of homology to both TE and flanking DNA. Thus our approach requires a minimal read length, which we find empirically to be greater than 65 bp. This limitation of minimal read length could be bypassed in principle by using paired-end data and attempting to assemble contigs that span the TE-flanking region junction. Third, we are not able to identify *de novo* insertions in repetitive regions of the genome (i.e. TE-rich pericentromeric regions) and thus many potential *de novo* TE insertion sites are not included in our data set. Despite these shortcomings, our approach has permitted the general properties of TIR and LTR element target sites in *D. melanogaster* to be generated in an automated and reproducible manner.

With the ability to generate a wealth of data on the natural target site properties for large numbers of TE families, genome-wide properties of TE target sites can now be uncovered in other species to test the generality of the conclusions reported here and further illuminate the molecular biology of transposition. Previous results from other species using classical approaches supports our ultimate conclusion that TSMs (which incorporate all lower level features of the data including position, orientation and TSD) are generally palindromic structures for TIR elements (see references in Table 1 of [6] and [17], [18], [49], [50]) and LTR elements/retroviruses [17], [51], [52], [53], [54], [55], [56]. Given the strong concordance between population genomic and classical data types in *D. melanogaster* (Table 4), we are confident that application of next-generation sequencing population genomics based methods to study TE target site properties will support this general finding across a wide range of species and TE families. Importantly, the common palindromic nature of TIR and LTR target sites suggest similar mechanisms for TIR and LTR insertion, which is supported by the fact that retroviral-like LTR elements use integrases that share catalytic activity with transposases of TIR elements [57]. Palindromic target sites are also generally consistent with transposases or integrases acting as multimeric complexes (e.g. [58], [59]), with the target site entering the catalytic complex along an axis of two-fold symmetry [60], [61]. Finally, the general AT-richness of TSMs may imply that flexibility of the target site sequence is crucial factor for the integration of many TE families [62]. These connections reveal how combining inferences from the rich natural resource of population genomic data with detailed structural and functional studies will benefit future work on the mechanistic basis of TE insertion into host genomes.

## Supporting Information

Figure S1 **DGRP Illumina experiments with unusual quality scores.** Boxplots of quality scores across the subset of Illumina reads that match the start or end of TE in the first stage of our mapping pipeline for the three DGRP strains with unusually low numbers of mapped TEs (SRS003443, SRS003447 and SRS003448) plus one strain representative of the typically quality score profile for the remainder of the strains sequence by Illumina (SRS003472).
(TIF)

Figure S2 **Frequency distribution of target site duplication lengths for** ***D. melanogaster*** **TE families.** Predicted TSD lengths for *de novo* TE insertions in the Illumina dataset for families with three or more insertion sites. The plots are organized by order (TIR then LTR) and superfamily, and are labeled with the order/superfamily, family name, predicted TSD length, and total number of insertions (in parentheses). All graphs have the same x-axis (from zero to 25 bp) with the y-axis varying according to the frequency of the elements. Sample sizes in this figure are based on individual insertion sites that can be present in more than one strain since each TSD is predicted independently.
(TIF)

File S1 **454 TE-genome junction reads.** UCSC Browser Extensible Format file with genomic locations of reads spanning TE-flanking genome junctions in strains of *Drosophila melanogaster* sequenced by the DGRP using 454 platform. The “name” field includes information about the family, order, SRA sample ID (SRS*), SRA run file ID (SRR*), and read ID in the indicated SRR file. Annotations are on are zero-based, half-open coordinate system relative to the Release 5 *D. melanogaster* genome sequence.
(TXT)

File S2 **Illumina TE-genome junction reads.** UCSC Browser Extensible Format file with genomic locations of reads spanning TE-flanking genome junctions in strains of *Drosophila melanogaster* sequenced by the DGRP using the Illumina platform. The “name” field includes information about the family, order, SRA sample ID (SRS*), SRA run file ID (SRR*), and read ID in the indicated SRR file. Annotations are on are zero-based, half-open coordinate system relative to the Release 5 *D. melanogaster* genome sequence.
(TXT)

File S3 **454 TE insertion sites.** UCSC Browser Extensible Format file with genomic locations of target sites of *de novo* insertions identified using the 454 platform. Coordinates represent the span of the target site duplication and the “score” field contains the total number of reads supporting that insertion site. Annotations are on are zero-based, half-open coordinate system relative to the Release 5 *D. melanogaster* genome sequence.
(TXT)

File S4 **Illumina TE insertion sites.** UCSC Browser Extensible Format file of genomic locations of target sites of *de novo* insertions identified using the Illumina platform. Coordinates represent the span of the target site duplication and the “score” field contains the total number of reads supporting that insertion site. Annotations are on are zero-based, half-open coordinate system relative to the Release 5 *D. melanogaster* genome sequence.
(TXT)

File S5 **Comparison of TE insertion sites for 25 strains with 454 and Illumina data.** Chromosome locations and presence/absence information for *de novo* TE insertions discovered in 25 strains from the DGRP project that were sequenced with both 454 and Illumina platforms.
(TXT)

File S6 **TSMs for 22** ***D. melanogaster*** **TE families.** Position frequency matrices for the TSM for 22 *D. melanogaster* TE families based on the Illumina platform.
(TXT)
