# Supplementary figures and images for: Whole Genome Resequencing Reveals Natural Target Site Preferences of Transposable Elements in Drosophila melanogaster
Source: PLoS One. 2012 Feb 9;7(2):e30008. doi: 10.1371/journal.pone.0030008 (PMC3276498; doi:10.1371/journal.pone.0030008)

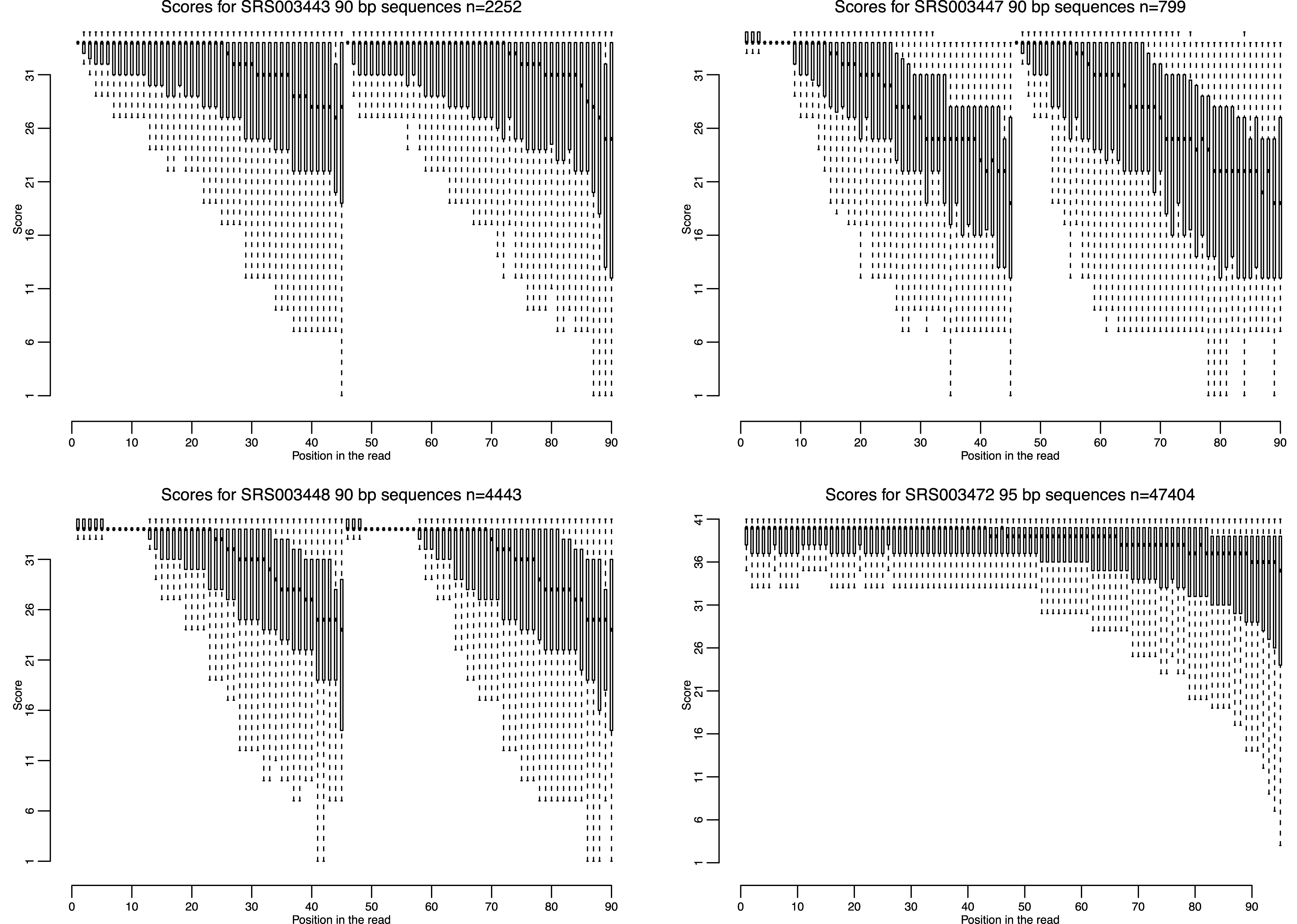

Supplement: Figure S1 — DGRP Illumina experiments with unusual quality scores. Boxplots of quality scores across the subset of Illumina reads that match the start or end of TE in the first stage of our mapping pipeline for the three DGRP strains with unusually low numbers of mapped TEs (SRS003443, SRS003447 and SRS003448) plus one strain representative of the typically quality score profile for the remainder of the strains sequence by Illumina (SRS003472). (TIF) [file pone.0030008.s001.tif]

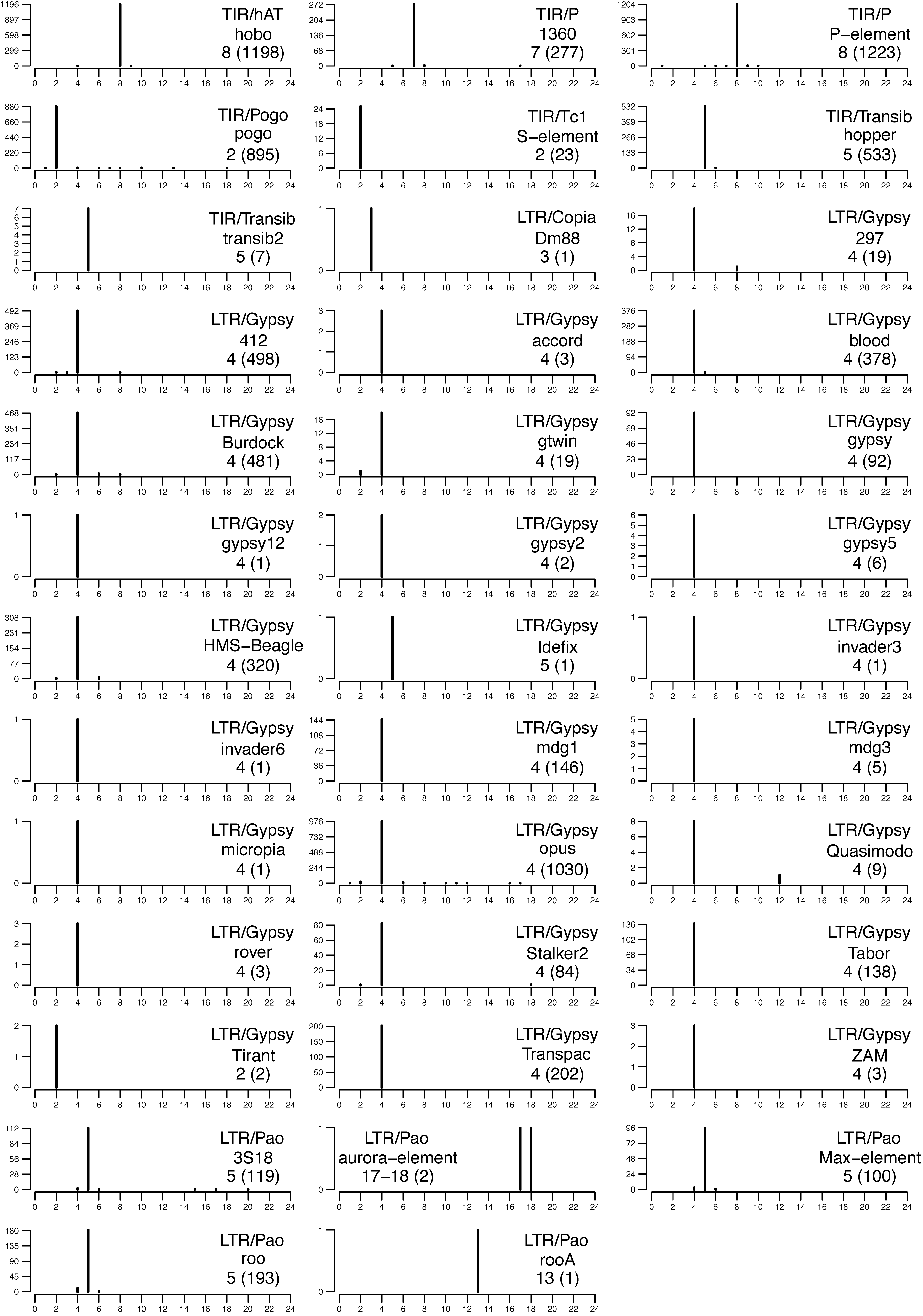

Supplement: Figure S2 — Frequency distribution of target site duplication lengths for D. melanogaster TE families. Predicted TSD lengths for de novo TE insertions in the Illumina dataset for families with three or more insertion sites. The plots are organized by order (TIR then LTR) and superfamily, and are labeled with the order/superfamily, family name, predicted TSD length, and total number of insertions (in parentheses). All graphs have the same x-axis (from zero to 25 bp) with the y-axis varying according to the frequency of the elements. Sample sizes in this figure are based on individual insertion sites that can be present in more than one strain since each TSD is predicted independently. (TIF) [file pone.0030008.s002.tif]
